# Heme oxygenase-1 activity is involved in the control of *Toxoplasma gondii* infection in the lung of BALB/c and C57BL/6 and in the small intestine of C57BL/6 mice

**DOI:** 10.1186/1297-9716-44-89

**Published:** 2013-10-02

**Authors:** Ester CB Araujo, Bellisa F Barbosa, Loyane B Coutinho, Paulo VC Barenco, Luciana A Sousa, Cristiane M Milanezi, Giuliano Bonfá, Wander R Pavanelli, João S Silva, Eloisa AV Ferro, Deise AO Silva, Jair P Cunha-Junior, Neide M Silva

**Affiliations:** 1 Laboratory of Immunopathology, Institute of Biomedical Sciences, Federal University of Uberlândia, Uberlândia, MG, Brazil; 2 Laboratory of Histology and Embriology, Institute of Biomedical Sciences, Federal University of Uberlândia, Uberlândia, MG, Brazil; 3 Department of Biochemistry and Immunology, Ribeirão Preto School of Medicine, University of São Paulo, Ribeirão Preto, SP, Brazil; 4 Department of Pathology Science, Biological Science Center, State University of Londrina, Londrina, PR, Brazil; 5 Laboratory of Immunoparasitology, Institute of Biomedical Sciences, Federal University of Uberlândia, Uberlândia, MG, Brazil

## Abstract

Heme oxygenase-1 (HO-1) is an enzyme that catabolizes free heme, which induces an intense inflammatory response. The expression of HO-1 is induced by different stimuli, triggering an anti-inflammatory response during biological stress. It was previously verified that HO-1 is able to induce indoleamine 2,3-dioxygenase (IDO), an enzyme that is induced by IFN-γ in *Toxoplasma gondii* infection. To verify the role of HO-1 during in vivo *T*. *gondii* infection, BALB/c and C57BL/6 mice were infected with the ME49 strain and treated with zinc protoporphyrin IX (ZnPPIX) or hemin, which inhibit or induce HO-1 activity, respectively. The results show that *T*. *gondii* infection induced high levels of HO-1 expression in the lung of BALB/c and C57BL6 mice. The animals treated with ZnPPIX presented higher parasitism in the lungs of both lineages of mice, whereas hemin treatment decreased the parasite replication in this organ and in the small intestine of infected C57BL/6 mice. Furthermore, C57BL/6 mice infected with *T*. *gondii* and treated with hemin showed higher levels of IDO expression in the lungs and small intestine than uninfected mice. In conclusion, our data suggest that HO-1 activity is involved in the control of *T*. *gondii* in the lungs of both mouse lineages, whereas the hemin, a HO-1 inducer, seems to be involved in the control of parasitism in the small intestine of C57BL/6 mice.

## Introduction

*Toxoplasma gondii* is an obligate intracellular parasite that infects humans and animals worldwide [1]. All mammals and birds that are consumed by humans may serve as intermediate hosts for *T*. *gondii* and, thus, may be a potential source of infection for humans [2]. In livestock, *T*. *gondii* tissue cysts are most frequently observed in various tissues of infected pigs, sheep and goats, and less frequently in infected poultry, rabbits, dogs and horses. By contrast, tissue cysts are found only rarely in skeletal muscles of cattle or buffaloes [3]. Although toxoplasmosis is a serious disease of humans, sheep, and many other warm-blooded animals [4], only a small percentage of exposed adult humans or animals develop clinical signs of the disease [5,6]. Toxoplasmosis is more severe in immunocompromised individuals and in cases of congenital infection [7]. The parasite is able to spread to all tissues and each tissue compartment has its own specific immune response [8].

Virtually all mouse lineages develop a Th1-type immune response to *T*. *gondii*, regardless of whether they present resistant or susceptible major histocompatibility complex (MHC) haplotypes [9,10]. However, a tight immunoregulation is necessary to prevent immunopathology [11-13]. Despite the type 1 immune response, C57BL/6 mice are more susceptible to the parasite than BALB/c mice [14-16]. The resistance to brain cyst burden and development of toxoplasmic encephalitis in mice were mapped to the regions D and L of MHC class I genes, more precisely the Ld (H-2^d^) region. Thus, mortality is substantially greater in mice that have H-2^b^ (C57BL/6) than in mice possessing an H-2d (BALB/c) background [17-20].

*T*. *gondii* infection induces IFN-γ production that is important to control parasite replication [21], and the toxoplasmacidal activity is associated to high levels of IFN-γ-triggered nitric oxide (NO) [22]. IFN-γ is also able to induce the enzyme indoleamine 2,3-dioxygenase (IDO), which degrades tryptophan, an essential amino acid for *T*. *gondii* growth [23]. During the early stage of *T*. *gondii* infection in the mouse model, IDO expression and tryptophan degradation are induced by endogenous IFN-γ [24,25].

Heme oxygenase (HO) is a stress-responsive enzyme that degrades free heme (iron protoporphyrin IX) to three products: gas carbon monoxide (CO), iron that induces the expression of heavy-chain (H-) ferritin (an iron-sequestering protein) and biliverdin, which is converted to bilirubin by biliverdin reductase (BVR) [26,27]. To date, three isoforms (HO-1, HO-2, and HO-3) that catalyze this reaction have been identified [24]. Under normal physiological conditions, most cells express low or undetectable levels of HO-1, while HO-2 is constitutively expressed [28,29].

The principal function of HO-1 is to avoid the accumulation of free heme [28], and HO-1 is inducible by numerous stimuli, such as NO, cytokines and growth factors, metalloporphyrins, hydrogen peroxide and lipid metabolites (reviewed in [30]). Although the function of this enzyme is still incompletely understood, previous studies suggest that the endogenous induction of HO-1 provides cytoprotective [31,32], anti-inflammatory [33,34] and anti-apoptotic [35] effects. The role of HO-1 is important in a broad range of inflammatory diseases, such as a sepsis model [36], airway inflammation [37] and virus infection [38]. HO-1 plays an important role in suppressing malaria pathology, since high expression of HO-1 is able to control susceptibility to cerebral malaria in mice [39]. From this aspect, BALB/c mice, which are less likely to develop experimental cerebral malaria (ECM) when infected with *Plasmodium berghei*, have a higher HO-1 expression than C57BL/6 mice, which develop ECM [39]. In contrast, HO-1 enzyme is necessary for the establishment of malaria liver stage; indeed HO-1 overexpression is associated with *P*. *berghei* liver infection, an effect that seems to be mediated by controlling the host inflammatory response [40]. Similarly, infections with *Leishmania pifanoi* parasites avoid the elicitation of superoxide production in infected macrophages by inducing HO-1 levels [41]. On the contrary, HO-1 expression seems to be involved in controlling parasitism in *Trypanosoma cruzi* infected mice [42].

The aim of the present study was to investigate the effect of HO-1 inhibition and induction during *T*. *gondii* infection in both resistant (BALB/c) and susceptible (C57BL/6) mouse lineages, considering the role of HO-1 during infectious diseases caused by protozoan parasites.

## Materials and methods

### Parasite strains

The ME49 strain of *T*. *gondii* was used to infect animals in this study. The strain was maintained in Swiss mice, which were inoculated by the intraperitoneal (i.p.) route with 20 cysts of *T*. *gondii*. A month after the inoculation, the brain cysts were collected and used to infect the animals.

For in vitro experiments, *T*. *gondii* tachyzoites of the 2 F1 strain, which constitutively express cytoplasmic β-galactosidase and are derived from the RH strain, were a gift from Dr Vern Carruthers, Medicine School of Michigan University (USA). The parasites were propagated in human uterine cervical (HeLa) cells obtained from the American Type Culture Collection (ATCC, Manassas, VA, USA) and maintained in RPMI 1640 medium (Cultilab, Campinas, SP, Brazil) supplemented with 100 U/mL penicillin, 100 μg/mL streptomycin (both reagents from Sigma Chemical Co., St. Louis, MO, USA) and 2% heat-inactivated fetal calf serum (FCS) (Cultilab) in a humidified incubator at 37 °C and 5% CO_2_.

### Animals

Female adult (8-12 week old) C57BL/6 and BALB/c mice were purchased from the School of Medicine of Ribeirão Preto, University of São Paulo, SP, Brazil and maintained under standard conditions in the Animal Experimentation Laboratory, Institute of Biomedical Sciences, Federal University of Uberlândia, MG, Brazil. All experimental procedures were approved by the Animal Experimental Ethics Committee (CEUA) of the Federal University of Uberlândia, with protocol number 007/09.

### Inhibition/induction of HO activity and *T*. *gondii* infection

ZnPPIX (Sigma) and hemin (Sigma) were used for inhibition and induction of the activity of HO, respectively. The reagents were dissolved in 50 mM Na_2_CO_3_ (pH 9.0) and the final volume was adjusted to an equal volume of 0.85% NaCl. C57BL/6 and BALB/c mice were treated subcutaneously with ZnPPIX (10 mg/kg/day), or hemin (5 mg/kg/day), or vehicle (Na_2_CO_3_ + NaCl - control group).

One day after the beginning of the treatment, C57BL/6 and BALB/c mice were infected with 5 cysts of *T*. *gondii* by the oral route as previously described [43-45] and were treated for an additional 11 days with ZnPPIX or hemin or vehicle. The animals were observed daily for morbidity and body weight changes. Morbidity scores were calculated as described elsewhere [46], as follows: sleek/glossy coat, bright and active (score 0); ruffled coat, hunched, tottering gait, reluctance to move (score 1); starry stiff coat (score 2). At 12 days post-infection (dpi), when the animals presented high morbidity scores, groups of six mice were anesthetized with ketamine (Syntec Brasil Ltda, Cotia, SP, Brazil) and Xylazine (Schering-Plough Coopers, Cotia, SP, Brazil) by the i.p. route and were killed by cervical dislocation. Blood samples were collected for serological assays and tissue samples, such as lung, liver, small intestine and brain, were collected, fixed in 10% buffered formalin, and processed routinely for paraffin embedding and sectioning or frozen immediately and stored in -80 °C for western blotting analysis.

### Determination of bilirubin levels in serum samples

In order to confirm the inhibition or induction of HO-1 activity in vivo, bilirubin, a secondary product of the enzyme activity, was measured in serum samples of treated mice by using an analytic kit (Labtest, Lagoa Santa, MG, Brazil) according to the manufacturer’s instructions. The absorbance was obtained at 520 nm and the bilirubin levels expressed in mg/dL.

### Quantification of tissue parasitism and HO-1 expression by immunohistochemistry

The parasitism and HO-1 expression was evaluated in the organs by immunohistochemistry as previously described [47]. Deparaffinized sections were incubated at room temperature with phosphate buffered saline (PBS) plus 3% non-fat milk (Nestle, São Paulo, SP, Brazil) to reduce nonspecific binding and then incubated at 4 °C overnight with polyclonal anti-*T*. *gondii* serum (obtained from *Calomys callosus* infected with ME49 strain) or goat anti-HO-1 antibody (Santa Cruz Biotechnology, Santa Cruz, CA, USA) both diluted in 0.01% saponin. After incubation with biotinylated goat anti-mouse antibody (Sigma) that recognizes *C*. *callosus* immunoglobulin or biotin-labeled donkey anti-goat antibody (Jackson ImmunoResearch Laboratories, West Grove, PA, USA), the assay sensitivity was improved by adding avidin-biotin-peroxidase complex (ABC kit, PK-4000; Vector Laboratories, Inc., Burlingame, CA, USA). The reaction was developed with 0.03% H_2_O_2_ plus 3,3′-diaminobenzidine tetrahydrochloride (DAB; Sigma) for 5 min. The sections were counterstained with Harris haematoxylin and examined under a light microscope using a 40× objective. The tissue parasitism was scored by counting the number of cyst-like structures and parasitophorous vacuoles from fifty microscopic fields in the lung and small intestine or per tissue section in the liver and brain in two histological sections of each mouse and from six mice per group.

### Cytokine (TNF-α, IFN-γ, TGF-β and IL-10), HO-1 and IDO mRNA expression in tissue samples by quantitative PCR (qPCR)

Fragments containing 100 mg were obtained from the lungs or ilea. RNA was extracted using an RNA extraction kit (Promega, Madison, WI, USA), according to the manufacturer’s instructions. The RNA concentration was determined (Biomate 3 spectrophotometer Thermospectronic, Rochester, NY, USA) and complementary DNA (cDNA) was synthesized using 1 μg of RNA through a reverse transcription reaction (M-MLV reverse transcriptase, Promega). Real-PCR quantitative mRNA analyses were performed on the ABI Prism 7500 Sequence Detection System using SYBR green fluorescence (Applied Biosystems, Warrington, UK). The standard PCR conditions were 95 °C for 10 min, 40 cycles for 1 min at 94 °C, 56 °C (1 min) and 72 °C (2 min), followed by a standard denaturation curve. The sequences of murine primers were designed using the Primer Express software (Applied Biosystems) and nucleotide sequences present in the GenBank database (Table 1). SYBR Green PCR Master Mix (Applied Biosystems), 0.1-0.2 μg/μL of specific primers, and 2.5 ng of cDNA were used in each reaction. The results were demonstrated as mRNA expression relative to non-infected mice. Calculations to determine the relative level of gene expression were made according to the instructions from Applied Biosystems User’s Bulletin #2 (P/N 4303859), with reference to β-actin in each sample, using the threshold cycle (C_t_) method. Negative controls without cDNA were also performed.

**Table 1:** Primer sequences used in real time PCR assays

**Primers**	**Sequences**
β-actin	Sense AGC TGC GTT TTA CAC CCT TT
	Antisense AAG CCA TGC CAA TGT TGT CT
TNF	Sense TGT GCT CAG AGC TTT CAA CAA
	Antisense CTT GAT GGT GGT GCA TGA GA
IFN-γ	Sense GCA TCT TGG CTT TGC AGC T
	Antisense CCT TTT TCG CCT TGC TGT TG
IL-10	Sense TGG ACA ACA TAC TGC TAA CC
	Antisense GGA TCA TTT CCG ATA AGG CT
TGF-β	Sense TGA ACC AAG GAG ACG GAA TAC A
	Antisense GGA GTT TGT TAT CTT TGC TGT CAC A
HO-1	Sense CCC AAA ACT GGC CTG TAA AA
	Antisense CGT GGT CAG TCA ACA TGG AT
IDO	Sense GGC AAA CTG GAA GAA AAA GG
	Antisense CAC CAG GAA ATG GAA CAG AAT G

### Detection of IDO protein in tissue homogenate by Western blotting

Fragments of lung and small intestine from BALB/c and C57BL/6 mice were dissected, washed in saline, frozen in liquid nitrogen, and stored at -80 °C until use. The tissue samples were pulverized in liquid nitrogen and promptly homogenized in extraction buffer (40 mM HEPES pH 7.7, 5 mM EDTA pH7.4, 1 mM benzamidine, 10 μg/mL aprotinin, 0.5 mM phenylmethane sulfonyl-fluoride, 2 mM dithiothreitol) and centrifuged at 15 000 × *g* for 5 min at 4 °C. The supernatants were collected, and the concentration of protein was measured by Bradford assay [48].

Protein samples (20 μg) were subjected to polyacrylamide gel electrophoresis under denaturing conditions (SDS-PAGE) at 10% and electrotransferred to PVDF membranes (Merck Millipore Headquarters, Billerica, MA, USA). Blotted membranes were incubated in blocking buffer (5% non-fat milk in 20 mM Tris buffered saline, pH 7.4 [TBS]) for 2 h at room temperature and incubated overnight with rat polyclonal anti-IDO antibody (Santa Cruz Biotechnology) or mouse monoclonal anti-β-actin antibody (Santa Cruz Biotechnology) at 1:200 or 1:1000 in TBS, respectively. Next, the membranes were exposed to peroxidase labeled goat anti-rat (Jackson ImmunoResearch Laboratories) diluted at 1:1000 for IDO detection or peroxidase labeled goat anti-mouse (Sigma) diluted at 1:3000 for β-actin detection in TBS with 1% non-fat milk, for 2 h at room temperature. The reaction was revealed by chemiluminescence (ECL kit, GE Healthcare, São Paulo, SP, Brazil) and an equal loading of the proteins was confirmed by staining the blots with 1% Ponceau. Densitometric analyses were performed by using the KODAK software (1D Image Analysis Software 3.5) in order to determine the mean intensity of the bands. The data were demonstrated as the relative density of the ratio between IDO and β-actin bands.

### *Toxoplasma gondii* proliferation in HeLa cells after pretreatment with ZnPPIX or hemin

In order to verify if ZnPPIX or hemin acts directly on the parasite, *T*. *gondii* proliferation in HeLa cells was analyzed. For this purpose, *T*. *gondii* 2 F1 tachyzoites (3 × 10^6^ parasites/well/0.2 mL) were incubated with RPMI medium containing Na_2_CO_3_ + NaCl (vehicle), ZnPPIX or hemin in different concentrations (1, 5, and 10 μM) for 1 h at 37 °C and 5% CO_2_. Next, parasites were centrifuged (720 × *g*, 5 min) and the resulting pellet was resuspended in RPMI medium with 2% FCS. The parasites were added to HeLa cell monolayer into 96-well plates (2 × 10^4^ cells/well/0.2 mL). After 24 h of infection, the cells were analyzed for *T*. *gondii* intracellular proliferation using the colorimetric β-galactosidase assay [49].

### Statistical analysis

Statistical analyses were carried out using GraphPad Prism 5 software (GraphPad Software, San Diego, CA, USA). Data were expressed as mean ± S.D. of experimental groups. The comparisons between C57BL/6 and BALB/c mice were analyzed by the Student’s *t* test, whereas comparisons between different experimental conditions within each mouse lineage were analyzed by one-way ANOVA and Bonferroni multiple comparison post-test. For in vitro experimental analyses, comparisons in relation to control were performed by one-way ANOVA and Dunnett post-hoc test. Differences were considered statistically significant when *P* < 0.05.

## Results

### *T*. *gondii* infection induces HO-1 activity predominantly in BALB/c mice

**Figure 1:**
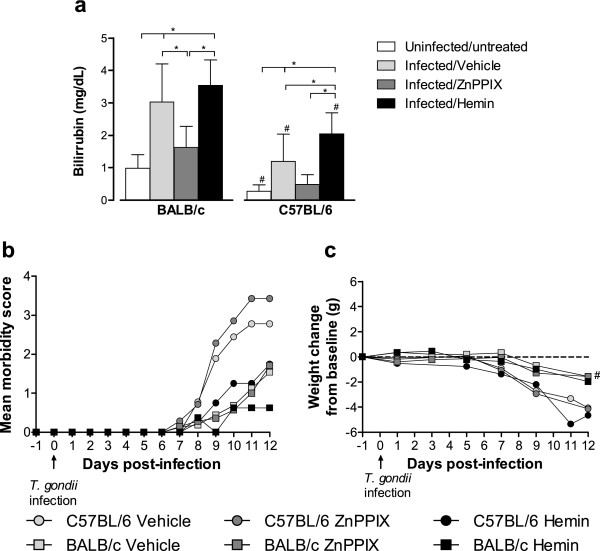
**Bilirubin serum levels (a), morbidity score (b), and body weight changes (c) of infected mice.** BALB/c and C57BL/6 mice were infected with 5 *T*. *gondii* cysts and treated with the inhibitor (ZnPPIX, 10 mg/kg/day) or inducer (hemin, 5 mg/kg/day) of HO-1 activity or vehicle (Na_2_CO_3_ + NaCl). Bilirubin levels were measured in sera of infected mice by an analytic kit as an indicative of HO-1 activity. Clinical parameters were observed daily until 12 dpi. Data are representative of at least two independent experiments of at least five mice per group. ^*^Statistically significant differences between different treatment conditions of BALB/c and C57BL/6 mice (ANOVA and Bonferroni multiple comparison post-test, *P* < 0.05). ^#^Statistically significant differences between the two mouse lineages submitted to the same treatment conditions (Student’s *t* test, *P* < 0.05).

In order to verify whether *T*. *gondii* infection is able to induce HO-1 activity, the bilirubin levels were measured in serum samples of BALB/c and C57BL/6 mice at 12 dpi. The inhibitory (ZnPPIX) or inducer (hemin) effect in the HO-1 activity was also analyzed. Infected BALB/c and C57BL/6 mice show higher serum bilirubin levels than uninfected/untreated controls (*P* < 0.01), indicating that *T*. *gondii* infection induced HO-1 activity (Figure 1a). The serum bilirubin levels decreased after treatment with ZnPPIX in both infected mouse lineages, although statistical significance was reached only in BALB/c mice (*P* < 0.01), indicating that the drug was able to reduce the HO-1 activity. When infected animals were treated with hemin, the bilirubin serum levels increased in comparison with uninfected/untreated mice (*P* < 0.01), indicating that the drug was able to increase the enzyme activity. However, hemin-treated BALB/c mice presented bilirubin levels similar to vehicle-treated animals, whereas hemin-treated C57BL/6 mice show increased bilirubin levels in comparison with their vehicle counterparts. In all experimental conditions, except for ZnPPIX treatment, C57BL/6 mice present lower serum bilirubin levels than BALB/c mice (*P* < 0.05; Figure 1a).

### Inhibition of HO-1 activity induces increased morbidity scores in *T*. *gondii*-infected C57BL/6 mice

The effect of the HO-1 activity during *T*. *gondii* infection was evaluated by clinical parameters as morbidity scores and body weight changes from baseline. Infected C57BL/6 mice showed more pronounced mean morbidity scores (Figure 1b) and significant body weight loss (*P* < 0.05; Figure 1c) in relation to BALB/c mice. C57BL/6 mice treated with ZnPPIX present increased morbidity scores as compared with vehicle- or hemin-treated animals or with BALB/c mice in all conditions, although statistical significance was not reached (Figure 1b). Also, the treatment with ZnPPIX or hemin shows no significant body weight changes among mice inside each lineage (Figure 1c).

### HO-1 activity is involved in the control of *T*. *gondii* infection mainly in the lung

**Figure 2:**
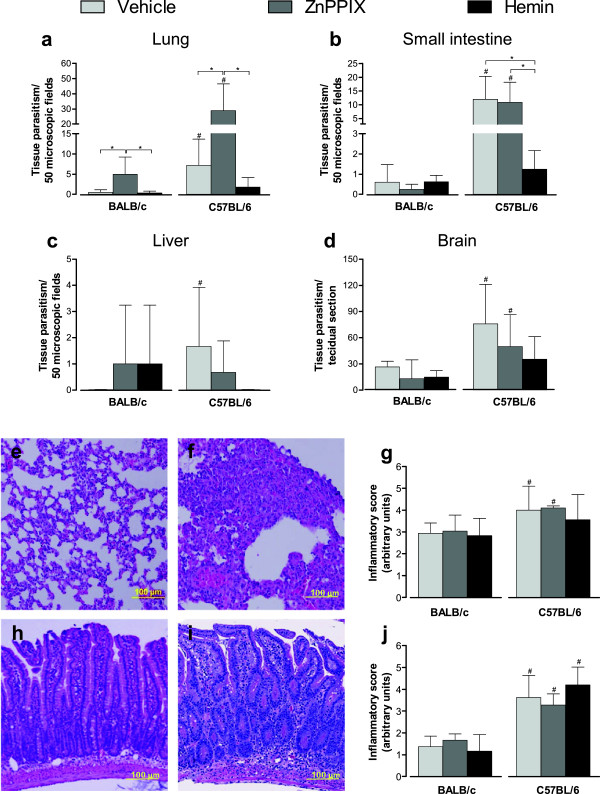
**Tissue parasitism and histological changes of infected BALB/c and C57BL/6 mice.** Both mice lineages were orally infected with 5 *T*. *gondii* cysts and treated with ZnPPIX (10 mg/kg/day), hemin (5 mg/kg/day) or vehicle (Na_2_CO_3_ + NaCl). The tissue parasitism in the lung **(a)**, small intestine **(b)**, liver **(c)** and brain **(d)** was detected by immunohistochemestry staining and scored by counting the number of parasitophorous vacuoles and cyst-like-structures per 50 microscopic fields in the lung and the small intestine or per tissue section in the liver and in the brain. Representative photomicrographs of histological changes of BALB/c and C57BL/6 mice **(e,f)** and inflammatory score in the lung **(g)** assessed by H&E staining. Representative photomicrographs of histological changes of BALB/c and C57BL/6 mice **(h,i)** and inflammatory score in the small intestine **(j)**. Data are representative of at least two independent experiments of five mice per group that provided similar results. ^*^Statistically significant differences between experimental conditions in the same lineage of mice (ANOVA and Bonferroni multiple comparison post-test, *P* < 0.05). ^#^Statistically significant differences between the two mouse lineages submitted to the same treatment conditions (Student’s *t* test, *P* < 0.05).

The tissue parasitism was assessed by determination of cyst-like structure and parasitophorous vacuole numbers in the peripheral organs and brain (Figure 2). On day 12 of *T*. *gondii* infection, C57BL/6 mice presented higher parasitism in the lung than BALB/c mice when treated with vehicle or ZnPPIX (*P* < 0.05; Figure 2a). ZnPPIX-treated C57BL/6 and BALB/c mice presented a significant increase in the parasite load in the lung (*P* < 0.01). The hemin treatment was able to decrease the parasitism in the lung of C57BL/6 mice related to vehicle-treated mice, despite not being statistically significant.

In the small intestine, C57BL/6 mice present higher parasitism compared with BALB/c mice when treated with vehicle or ZnPPIX (*P* < 0.05; Figure 2b). The induction or reduction of HO-1 activity did not interfere with parasite control in this organ in BALB/c mice. In C57BL/6 mice, however, the induction of the enzyme activity by hemin treatment was able to decrease parasite load in the small intestine (*P* < 0.01), whereas the reduction of HO-1 activity by ZnPPIX treatment did not alter the parasite burden in this organ (Figure 2b).

In the liver, BALB/c and C57BL/6 mice showed low parasite burden regardless of HO-1 activity (Figure 2c). However, vehicle-treated C57BL/6 mice present higher liver parasitism than vehicle-treated BALB/c mice (*P* < 0.05; Figure 2c). HO-1 activity did not alter the parasite burden in the brain of BALB/c or C57BL/6 mice on 12 dpi (Figure 2d), and C57BL/6 mice present higher brain tissue parasitism compared with BALB/c mice when treated with vehicle or ZnPPIX (*P* < 0.05; Figure 2d).

The histological changes in the lung and small intestine of infected mice were also investigated, since these organs presented higher tissue parasitism on 12 dpi. The lung of BALB/c and C57BL/6 mice present lesions that were constituted by inflammatory exudates of mononucleated cells within the alveolar walls, enlarging the pulmonary septum (Figure 2e, Figure 2f). The lesions were significantly higher in the lung of C57BL/6 compared with BALB/c mice (*P* < 0.05; Figure 2i). On 12 dpi, the small intestine of C57BL/6 mice presented lesions that were characterized by infiltration of mononuclear cells into lamina propria (LP), epithelium and submucosa (Figure 2g). BALB/c mice presented smaller inflammatory changes in the organ compared with C57BL/6 mice in the same experimental condition (*P* < 0.05; Figure 2g, Figure 2h, Figure 2j). In some areas of the small intestine of C57BL/6 mice, increased thickness of the intestinal villi was observed. Interestingly, the inhibition or induction of HO-1 did not significantly alter the severity of the lesions in comparison with vehicle-treated and infected mice within the same lineage (Figure 2j).

### *T*. *gondii* infection increases IFN-γ, IL-10 and IDO mRNA expression in the lung of C57BL/6 mice

**Figure 3:**
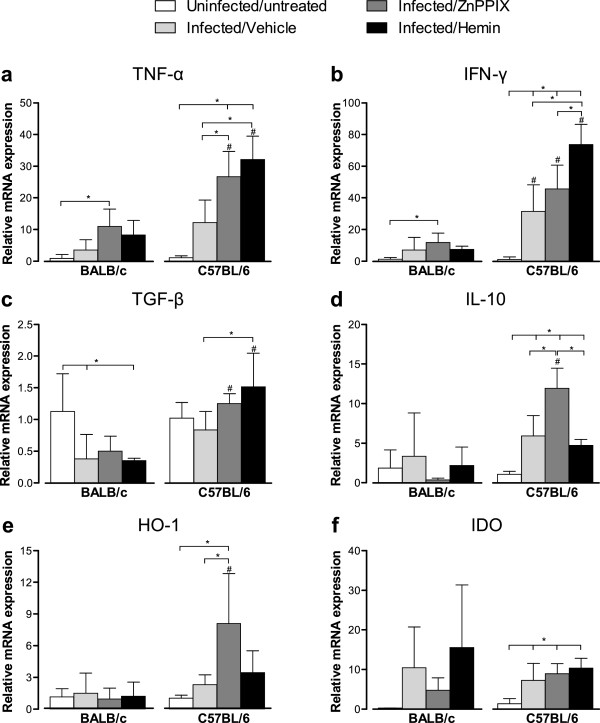
**Cytokines, HO-1 and IDO mRNA expression in the lung of *T*. *gondii *infected mice.** BALB/c and C57BL/6 mice were infected with 5 *T*. *gondii* cysts by oral route and treated with ZnPPIX (10 mg/kg/day), hemin (5 mg/kg/day) or vehicle (Na_2_CO_3_ + NaCl) and analyzed at 12 dpi. The relative levels of TNF-α **(a)**, IFN-γ **(b)**, TGF-β **(c)**, IL-10 **(d)**, HO-1 **(e)** and IDO **(f)** gene expression were calculated by reference to the β-actin in each sample, using the threshold cycle (C_t_) method. Data are representative of at least two independent experiments of five mice per group. ^*^Statistically significant differences between experimental conditions in the same lineage of mice (ANOVA and Bonferroni multiple comparison post-test, *P* < 0.05). ^#^Statistically significant differences between the two mouse lineages submitted to the same treatment conditions (Student’s *t* test, *P* < 0.05).

As HO-1 activity seems to be involved in the control of parasite burden in the lung of BALB/c and C57BL/6 mice, expression of TNF-α, IFN-γ, TGF-β and IL-10 mRNA was measured in this site at 12 dpi (Figure 3a-d). In addition, HO-1 and IDO mRNA expression was measured in infected mice treated with ZnPPIX or hemin (Figure 3e, Figure 3f). *T*. *gondii* infection was able to increase pulmonary IFN-γ, IL-10 and IDO messages in C57BL/6 mice and to decrease pulmonary TGF-β mRNA expression in BALB/c mice (*P* < 0.05). The ZnPPIX treatment induced higher TNF-α and IFN-γ mRNA expression in both mouse lineages, and higher IL-10, HO-1 and IDO mRNA expression in C57BL/6 mice in comparison with uninfected/untreated controls (*P* < 0.05). When compared with vehicle-treated infected mice, the ZnPPIX treatment induced higher TNF-α, IL-10 and HO-1 mRNA expression in C57BL/6 mice only (*P* < 0.05). TGF-β mRNA expression was not significantly changed with the ZnPPIX treatment in both mouse lineages. The hemin treatment induced higher TNF-α, IFN-γ, IL-10 and IDO mRNA expression in C57BL/6 mice and lower TGF-β mRNA expression in BALB/c mice, when compared with uninfected/untreated controls (*P* < 0.05). Also, hemin-treated C57BL/6 >mice show increased TNF-α, IFN-γ and TGF-β mRNA expression in comparison with the vehicle treatment, and increased IFN-γ and decreased IL-10 mRNA expression in comparison with ZnPPIX treatment (*P* < 0.05). HO-1 mRNA expression was not significantly changed with the hemin treatment in both mouse lineages.

When comparing both mouse lineages, C57BL/6 mice present higher mRNA expression of all analyzed cytokines and HO-1 than BALB/c mice, when treated with ZnPPIX or hemin (*P* < 0.05). IDO mRNA expression in C57BL/6 mice was not changed with the treatments and was similar to that of BALB/c mice.

### *T*. *gondii* infection does not induce significant changes in the HO-1 or IDO mRNA expression in the small intestine of BALB/c or C57BL/6 mice

Since the induction of HO-1 activity by hemin treatment was able to reduce parasite replication in the small intestine of C57BL/6 mice, we investigated the mRNA expression of cytokines (TNF-α, IFN-γ, TGF-β and IL-10) and enzymes (HO-1 and IDO) in the small intestine of *T*. *gondii*-infected animals treated with ZnPPIX or hemin.

**Figure 4:**
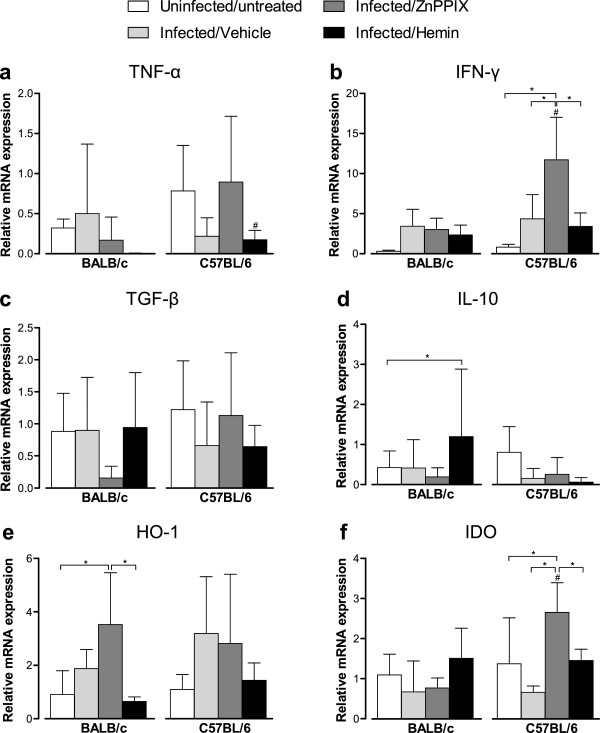
**Cytokines, HO-1 and IDO mRNA expression in the small intestine of *T*. *gondii *infected mice.** BALB/c and C57BL/6 mice were infected with 5 *T*. *gondii* cysts by oral route and treated with ZnPPIX (10 mg/kg/day), hemin (5 mg/kg/day) or vehicle (Na_2_CO_3_ + NaCl) and analyzed at 12 dpi. The relative levels of TNF-α **(a)**, IFN-γ **(b)**, TGF-β **(c)**, IL-10 **(d)**, HO-1 **(e)** and IDO **(f)** gene expression were calculated by reference to the β-actin in each sample, using the threshold cycle (C_t_) method. Data are representative of at least two independent experiments of five mice per group. ^*^Statistically significant differences between experimental conditions in the same lineage of mice (ANOVA and Bonferroni multiple comparison post-test, *P* < 0.05). ^#^Statistically significant differences between the two mouse lineages submitted to the same treatment conditions (Student’s *t* test, *P* < 0.05).

*T*. *gondii* infection was not able to change significantly the cytokine and enzyme messages in the small intestine in both mouse lineages on 12 dpi (Figure 4). When animals were treated with ZnPPIX, however, C57BL/6 mice show higher IFN-γ and IDO mRNA expression as compared with uninfected/untreated or vehicle-treated infected animals (*P* < 0.05). On the contrary, ZnPPIX-treated BALB/c mice show increased HO-1 mRNA expression in comparison with uninfected/untreated animals (*P* < 0.05). The hemin treatment of BALB/c mice induced higher IL-10 mRNA expression compared with uninfected/untreated mice (*P* < 0.05) and decreased HO-1 mRNA expression in relation to ZnPPIX-treated animals (*P* < 0.05). In C57BL/6 mice, the hemin treatment induced a decrease of IFN-γ and IDO mRNA expression compared with ZnPPIX-treated animals (*P* < 0.05). When the mouse lineages were compared, C57BL/6 mice show higher TNF-α mRNA expression than BALB/c when treated with hemin, and higher IFN-γ and IDO mRNA expression when treated with ZnPPIX (*P* < 0.05).

### HO-1 expressing cells are increased in the lung of BALB/c and C57BL/6 mice during *T*. *gondii* infection

**Figure 5:**
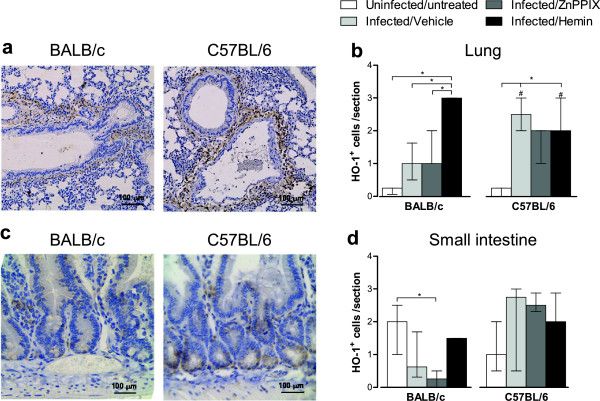
**Immunoperoxidase staining of HO-1 in the lung and small intestine of *T*. *gondii *infected mice.** BALB/c and C57BL/6 mice were orally infected with 5 *T*. *gondii* cysts and treated with ZnPPIX (10 mg/kg/day), hemin (5 mg/kg/day) or vehicle (Na_2_CO_3_ + NaCl) and analyzed at day 12 pi. Representative photomicrographs of lung of hemin-treated mice **(a)** and small intestine of untreated/uninfected mice **(c)**. The images were captured using a 10 × objective. The data were obtained by counting the number of HO-1 positive cells per tissue section of the lung **(b)** and small intestine **(d)** using a 40 × objective. Data represent the median with interquartile range of each experimental condition in two independent experiments. ^*^Statistically significant differences between the experimental conditions within the same mouse lineage (*P* < 0.05, Kruskal Wallis test and Dunn multiple comparison post-test); ^#^Statistically significant differences between the two mouse lineages (*P* < 0.05, Mann–Whitney test).

HO-1 expressing cells (HO-1^+^) were determined by immunohistochemistry in pulmonary and intestinal tissues of infected BALB/c and C57BL/6 mice. *T*. *gondii* infection increased the number of HO-1^+^ cells in the lungs of BALB/c and C57BL/6 mice compared with untreated/uninfected mice, although with statistical significance only in C57BL/6 mice (*P* < 0.05; Figure 5a, Figure 5b). The treatment with ZnPPIX did not alter the amount of HO-1^+^ cells in the lung and small intestine of both mouse lineages. On the contrary, the hemin treatment of BALB/c mice induced higher numbers of HO-1^+^ cells compared with untreated/uninfected controls or vehicle- or ZnPPIX-treated BALB/c mice (*P* < 0.05). Hemin-treated C57BL/6 mice show lower number of HO-1^+^ cells compared with BALB/c mice (*P* < 0.05), whereas vehicle-treated C57BL/6 mice present higher HO-1^+^ cells than BALB/c mice under the same conditions (*P* < 0.05; Figure 5b).

In the small intestine, *T*. *gondii* infection did not alter the number of HO-1^+^ cells in both mouse lineages, but the ZnPPIX treatment reduced HO-1^+^ cells in infected BALB/c mice as compared with untreated/uninfected controls (*P* < 0.05; Figure 5c, Figure 5d).

### *T*. *gondii* infection increases the IDO expression in the lungs and small intestine mainly in C57BL/6 mice

**Figure 6:**
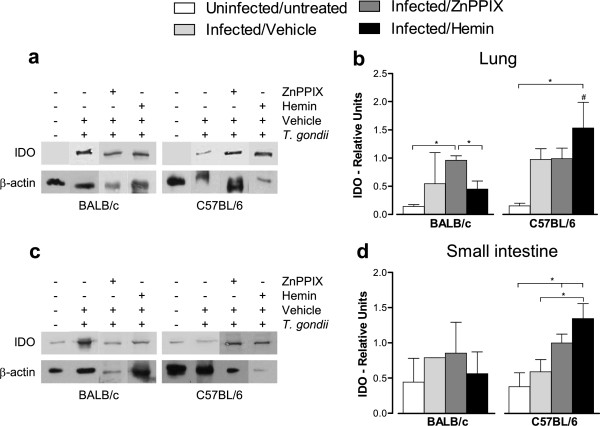
**Detection of IDO in the lung (a,b) and small intestine (c,d) of *T*. *gondii *infected mice.** BALB/c and C57BL/6 mice were orally infected with 5 *T*. *gondii* cysts and treated with ZnPPIX (10 mg/kg/day), hemin (5 mg/kg/day) or vehicle (Na_2_CO_3_ + NaCl) and analyzed at day 12 pi. Protein extracts obtained from tissue samples were submitted to Western blotting assay for detection of IDO expression. Representative Western blotting for IDO and β-actin in the lung **(a)** and small intestine **(c)** and densitometric analyses showing the relative density of the respective proteins obtained by the ratio between IDO/β-actin bands in the lung **(b)** and small intestine **(d)**. Data are representative of at least two independent experiments of five mice per group. ^*^Statistically significant differences between experimental conditions in the same lineage of mice (ANOVA and Bonferroni multiple comparison post-test, *P* < 0.05). ^#^Statistically significant differences between the two mouse lineages submitted to the same treatment conditions (Student’s *t* test, *P* < 0.05).

In order to verify the effect of *T*. *gondii* infection and HO-1 activity in the IDO protein expression in the lungs and small intestine of mice, tissue samples obtained on 12 dpi were submitted to western blot analyses. Both mouse lineages presented a trend to higher IDO levels in the lungs compared with untreated/uninfected controls (Figure 6a, Figure 6b). ZnPPIX-treated BALB/c mice presented higher levels of IDO protein compared with uninfected/untreated controls and hemin-treated animals (*P* < 0.05; Figure 6b). On the contrary, when analyzing infected C57BL/6 mice, the hemin treatment induced higher IDO protein expression in the lungs compared with untreated/uninfected controls and BALB/c mice in the same condition (*P* < 0.05; Figure 6b).

The IDO protein expression was also measured in the small intestine of mice and it was verified that the infection did not induce IDO in the small intestine of BALB/c mice, irrespective that the animals were treated or not with ZnPPIX or hemin (Figure 6c, Figure 6d). However, hemin treatment of infected C57BL/6 mice induced higher IDO protein expression as compared with untreated/uninfected controls or vehicle- or ZnPPIX-treated mice (*P* < 0.05; Figure 6c, Figure 6d).

### ZnPPIX and hemin do not act directly on *T*. *gondii*

To determine whether ZnPPIX and hemin could interfere in *T*. *gondii* replication, parasites were treated with the drugs before infection of Hela cells. An additional figure file shows that parasites previously treated with different concentrations of ZnPPIX or hemin presented similar proliferation in relation to parasites pretreated with vehicle only. In addition, comparison between the drugs shows that *T*. *gondii* proliferation was similar in both treatments (see Additional file 1).

## Discussion

The HO-1 enzyme is induced in many inflammatory disorders caused by protozoan parasites, such as *L*. *chagasi*[50], *L*. *pfanoi*[41], *Plasmosdium chabaudi*[51], *P*. *berghei*[39] and *T*. *cruzi*[42]. Thus, we were interested in knowing whether HO-1 could also participate in the immune response to *T*. *gondii* infection. Our results show that the HO-1 activity is enhanced during experimental toxoplasmosis, as demonstrated by the higher serum bilirubin levels in infected mice as compared to uninfected controls. Interestingly, HO-1 activity was higher in BALB/c mice, a lineage considered more resistant to chronic *T*. *gondii* infection compared with the susceptible C57BL/6 lineage. On the contrary, inhibition of HO-1 activity by ZnPPIX treatment induced more pronounced morbidity scores and body weight loss in the susceptible rather than resistant mouse lineage. Accordingly, in our previous study [47], we demonstrated that *T*. *gondii* infection induces higher inflammatory lesions in peripheral organs of C57BL/6 compared with BALB/c mice. It is noteworthy that in the present study, we used a low dose of infection (5 cysts of the ME49 strain) and stopped the experiment at 12 days post-infection, when the animals, particularly of the C57BL/6 mouse lineage, had high morbidity scores. *T*. *gondii* experimental infections in different mouse lineages used doses ranging from 1 to 100 cysts of different strains (P-Br and ME49) of *T*. *gondii*[43,44,52]. In these studies, a reduction in survival starting from the 8^th^-10^th^ day post-infection and increased inflammatory changes even with the lowest dose used in the highly susceptible mouse lineage (C57BL/6), as we observed in our investigation, was demonstrated. Thus, we decided to use 5 *T*. *gondii* cysts to infect animals.

Since the anti-inflammatory effects of HO-1 have been extensively characterized [53], we hypothesized that the inhibition of HO-1 activity by ZnPPIX treatment would increase inflammatory changes in *T*. *gondii* infection, while the enzyme induction by hemin would attenuate the histological changes induced by the parasite. Unexpectedly, the induction or inhibition of HO-1 activity at levels obtained in our experimental procedure did not alter the inflammatory lesions in the organs of both BALB/c and C57BL/6 mice at 12 dpi. Previous studies in experimental cerebral malaria demonstrated that the inhibition of HO-1 or HO-1 deletion in resistant BALB/c mice led to disruption of the blood brain barrier and brain microvascular congestion with activated leukocytes and red blood cells [39]. In susceptible C57BL/6 mice, the induction of HO-1 by cobalt protoporphyrin-IX (CoPPIX) reduced blood brain barrier disruption and abolished brain microvascular congestion [39]. Likewise, during *Plasmodium* infection, the liver of Hmox^-/-^ (BALB/c or C57BL/6) mice presented increased number and size of inflammatory foci and proinflammatory cells compared with Hmox^+/+^ mice [40], and HO-1 expression in the liver was associated with decreased necrosis in DBA/2 mice [51]. These results reflect that different protozoan parasites induce diverse immune response and, in the case of *T*. *gondii* infection, the HO-1 activity did not interfere with the inflammatory alterations induced by the parasite.

The HO-1 inhibition by ZnPPIX treatment induced higher parasitism in the lungs at 12 dpi in both mouse lineages compared with hemin or vehicle treatment, although the parasitism was higher in C57BL/6 than BALB/c mice. The treatment with hemin was able to decrease the parasite load in the lung of C57BL/6 mice. Also, *T*. *gondii* infection induced increased amounts of HO-1^+^ cells in the lung of both mouse lineages, predominantly in C57BL/6 mice. In the small intestine, high parasite load was seen in C57BL/6 mice and the HO-1 induction by hemin treatment was able to decrease the parasitism. Interestingly, this effect was not due to direct action on the parasite, since in vitro experiments showed that *T*. *gondii* proliferation was not changed after parasite pretreatment with hemin or ZnPPIX. Similar results were found in *T*. *cruzi*-infected C57BL/6 mice, since the HO-1 induction by CoPPIX reduced parasitemia and tissue parasitism [42]. On the contrary, HO-1 overexpression in mice induced an increase in *P*. *burghei* liver burden, and treatment of mice with CO, an end-product of HO-1 enzymatic activity, enhanced parasite burden, suggesting that the anti-inflammatory properties of HO-1 were favorable to this parasite replication [40]. Another study demonstrated that ZnPPIX treatment did not alter parasite replication in experimental cerebral malaria [39].

Related to cytokine mRNA expression in the lung and small intestine, it was observed that *T*. *gondii*-infected C57BL/6 mice presented higher levels of TNF-α and IFN-γ expression in the lung on 12 dpi compared with BALB/c mice as previously shown [47]. These data were in accordance with previous studies that showed high serum levels of pro-inflammatory cytokines, such as IFN-γ, TNF-α and IL-6 in infected BALB/c mice [54]. The increased production of pro-inflammatory cytokines in C57BL/6 mice could be related to intense histological changes and accentuated clinical parameters. Accordingly, C57BL/6 mice infected orally with the 76 K strain of *T*. *gondii* showed higher serum levels of TNF-α and IFN-γ at 9 dpi and increased weight loss compared with BALB/c mice [55]. Recently, it was demonstrated that HO-1 induction by treatment with CoPPIX did not alter the production of IFN-γ by splenocytes stimulated with *T*. *cruzi* antigen at 9 dpi [42]. Our results show that the drugs have a different effect in *T*. *gondii*-infected organs; the ZnPPIX or hemin treatment increased TNF-α and IFN-γ expressions in the lung of infected C57BL/6 mice whereas the hemin treatment diminished the parasite load in the organ, hypothesizing that the mechanisms controlling the parasite induced by hemin are independent of these two cytokines. Regarding the small intestine, the HO-1 activity did not interfere with the expression of pro-inflammatory cytokines in BALB/c mice on 12 dpi. Conversely, ZnPPIX-treated C57BL/6 mice presented higher levels of IFN-γ expression in the organ in relation to the other treatments, but this elevated cytokine expression was not sufficient to control the parasite in the organ.

Previous studies have reported that spleen cells from *T*. *gondii*-infected mice produce IL-10 at 7 dpi [56]. Moreover, TGF-β and IL-10 mRNA levels were higher in the peripheral lymph node cells of heart transplanted mice treated with CoPPIX, and treatment with HO-1 inhibitor (ZnPPIX) decreased IL-10 expression in these animals [57]. In the present study, *T*. *gondii* infection induced higher levels of IL-10 mRNA in the lung of C57BL/6 mice, with no difference in the small intestine. The ZnPPIX treatment increased the IL-10 mRNA expression in the lung of C57BL/6 mice. *T*. *gondii* infection did not alter significantly TGF-β expression in the lungs of C57BL/6, but decreased its expression in the lungs of BALB/c mice; and no alteration was observed in the small intestine compared with uninfected mice in the two lineages. Since there was no difference in histological changes among mice infected and treated or not with ZnPPIX/hemin, we suggest that IL-10 and TGF-β are not involved in the control of pathology or parasitism in the lungs and small intestine of infected mice in these experimental conditions. Accordingly, Paiva et al. [42] demonstrated that conventional *T*. *cruzi* elimination mechanisms are not involved in the reduction of parasitism induced by CoPPIX.

Previous studies have demonstrated that the hemin treatment increases IDO and HO-1 protein expression in bone marrow-derived dendritic cells and the ZnPPIX treatment abrogated IDO expression in LPS-stimulated dendritic cells in vivo and in vitro [58]. It is well known that restriction of available tryptophan due to degradation by IDO leads to the control of *T*. *gondii* replication in vitro [23,59,60]. Additionally, IDO inhibition during toxoplasmosis led to 100% mortality, with increased parasite burden in the brain [61]. In this context, we investigated the HO-1 mRNA expression in the lung and observed that the infection induced HO-1 expression in the organ of C57BL/6 mice, and higher IDO expression than uninfected mice in both mouse lineages. Interestingly, ZnPPIX treatment induced higher HO-1 mRNA expression in the lungs of C57BL/6 mice compared with uninfected or vehicle-treated infected mice. Since ZnPPIX inhibits HO-1 activity competitively [62], an enhanced HO-1 transcription could be a cellular mechanism in response to low levels of functional HO-1 protein.

Previous investigations demonstrated high levels of IDO expression and tryptophan degradation in the lungs of C57BL/6 mice during acute *T*. *gondii* infection [24,25]. However, in our investigation, the HO-1 activity did not alter the IDO mRNA expression in the lungs of infected mice from both lineages, although higher levels of IDO protein were observed in C57BL/6 mice under hemin treatment. This additional induction of IDO by hemin in the lungs of infected mice, above that is normally induced by infection, seemed to be involved in the control of the parasite in C57BL/6 mice. Regarding the resistant BALB/c mice, our findings suggest that baseline levels of HO-1 protein are required for the control of parasitism in the lung, since HO-1 inhibition increased *T*. *gondii* replication. However, this mechanism seems to be independent of IDO, as demonstrated by higher IDO protein expression in the lung of BALB/c mice treated with ZnPPIX. In this context, the exact role of HO-1 in this process remains to be elucidated.

Although IDO expression in the small intestine has not been completely elucidated during *T*. *gondii* infection, other studies have demonstrated that IDO is important during inflammatory responses in this site reviewed by [63]. This enzyme has a protective role in *Citrobacter rodentium* enteric bacterial pathogen infection [64]. When analyzing the small intestine of BALB/c mice, our results suggest that HO-1 and IDO mechanisms are not crucial to control *T*. *gondii* in this resistant mouse lineage. Conversely, our results show that treatment with hemin, despite not interfering with this enzyme mRNA expression, induces high levels of IDO protein in the organ of C57BL/6 mice, and reduced *T*. *gondii* replication. Interestingly, HO-1 expression was similar in all treatment conditions. However, bilirubin levels indicate that HO-1 activity is increased in mice treated with hemin, suggesting that the mechanisms involved in controlling *T*. *gondii* growth in the small intestine are at least partially associated with HO-1 activity.

Taken together, our data suggest that HO-1 activity is involved in the control of *T*. *gondii* infection in the lung of BALB/c and C57BL/6 mice; and hemin, a HO-1 inducer can be involved at least in part in the control of parasite load in the small intestine of C57BL/6 mice. However, the exactly HO-1-mediated mechanisms of controlling *T*. *gondii* in the lung and small intestine, two important sites of infection, are incompletely known and remain to be established.

## Abbreviations

HO-1: Heme oxygenase-1; IDO: Indoleamine 2,3-dioxygenase; NO: Nitric oxide; ZnPPIX: Zinc protoporphyrin IX.

## Competing interests

The authors declare that they have no competing interests.

## Authors’ contributions

ECBA was an MS student and was involved in parasite strain maintenance in vivo and in cell culture, in ZnPPIX and hemin treatment and *Toxoplasma gondii* infection of the animals, serum biochemical analysis, indoleamine-2,3-dioxygenase detection by western blotting. BFB was a PhD collaborator and was involved in western blotting assays and with experiments with cell culture. LBC, LAS and PVCB were MS students that were involved in care with the animals and daily observation for clinical signs and mortality, and also with the immunohistochemistry assays. CMM and GB were MS and JSS was a PhD collaborator and both were involved in qPCR assays. WRP was a PhD collaborator and was involved in the experimental design. EAVF and JPCJ were PhD collaborators and were involved in the western blotting assays, experimental design, data analysis and revision of the manuscript. DAOS was a PhD collaborator and was involved in the experimental design, data and statistical analysis and revision of the manuscript. NMS was a PhD researcher and was involved in the study design, interpretation of results, statistical analysis and had final responsibility for the study. All authors read and approved the manuscript.

## Supplementary Material

Additional file 1 ***T gondii *intracellular proliferation in HeLa cells after parasite treatment with ZnPPIX, hemin or vehicle.** Tachyzoites of *T*. *gondii* 2 F1 strain were treated for 1 h with different concentrations of ZnPPIX or hemin or vehicle. After 24 h of infection, the experiment was analyzed for *T*. *gondii* intracellular proliferation determined by a colorimetric microtiter assay using β-galactosidase-expressing tachyzoites. Data are expressed as mean ± SD of the number of tachyzoites calculated in relation to a reference curve and are representative of two independent experiments performed in quadruplicate. Click here for file

## References

[B1] Rorman E Zamir CS Rilkis I Bem-David H Congenital toxoplasmosis – prenatal aspects of *Toxoplasma gondii* infection Reprod Toxicol 2006 21 458 472 16311017 10.1016/j.reprotox.2005.10.006

[B2] Tenter AM *Toxoplasma gondii* in animals used for human consumption Mem Inst Oswaldo Cruz 2009 104 364 369 19430665 10.1590/s0074-02762009000200033

[B3] Tenter AM Heckeroth AR Weiss LM *Toxoplasma gondii*: from animals to humans Int J Parasitol 2000 30 1217 1258 11113252 10.1016/s0020-7519(00)00124-7 PMC3109627

[B4] Dubey JP Schares G Neosporosis in animals–the last five years Vet Parasitol 2011 180 90 108 21704458 10.1016/j.vetpar.2011.05.031

[B5] Montoya JG Liesenfeld O Toxoplasmosis Lancet 2004 363 1965 1976 15194258 10.1016/S0140-6736(04)16412-X

[B6] Dubey JP Jones JL *Toxoplasma gondii* infection in humans and animals in the United States Int J Parasitol 2008 38 1257 1278 18508057 10.1016/j.ijpara.2008.03.007

[B7] Remington JS McLeod R Thulliez P Desmonts G Remington JS, Klein J Toxoplasmosis Infection diseases of the fetus and newborn infant 2001 5 Philadelphia: Saunders 205 356

[B8] Filisetti D Candolfi E Immune response to *Toxoplasma gondii* Ann Ist Super Sanita 2004 40 71 80 15269455

[B9] Gazzinelli RT Hakim FT Hieny S Shearer GM Sher A Synergistic role of CD4^+^ and CD8^+^ T lymphocytes in IFN-γ production and protective immunity induced by an attenuated *Toxoplasma gondii* vaccine J Immunol 1991 146 286 292 1670604

[B10] Gazzinelli RT Hu Y Hieny S Cheever A Sher A Simultaneous depletion of CD4^+^ and CD8^+^ T lymphocytes is required to reactive chronic infection with *Toxoplasma gondii* J Immunol 1992 149 175 180 1351500

[B11] Gazzinelli RT Wysocka M Hieny S Scharton-Kersten T Cheever A Kühn R Müller W Trinchieri G Sher A In the absence of endogenous IL-10, mice acutely infected with *Toxoplasma gondii* succumb to a lethal immune response dependent on CD4^+^ T cells and accompanied by overproduction of IL-12, IFN-gamma and TNF-alpha J Immunol 1996 157 798 805 8752931

[B12] Neyer LE Grunig G Fort M Remington JS Rennick D Hunter CA Role of interleukin-10 in regulation of T-cell-dependent and T-cell-independent mechanisms of resistance to *Toxoplasma gondii* Infect Immun 1997 65 1675 1682 9125546 10.1128/iai.65.5.1675-1682.1997 PMC175195

[B13] Suzuki Y Sher A Yap G Park D Neyer LE Liesenfeld O Fort M Kang H Gufwoli E IL-10 is required for prevention of necrosis in the small intestine and mortality in both genetically resistant BALB/c and susceptible C57BL/6 mice following peroral infection with *Toxoplasma gondii* J Immunol 2000 164 5375 5382 10799901 10.4049/jimmunol.164.10.5375

[B14] McLeod R Skamene E Brown C Eisenhauer PB Mack D Mack DG Genetic regulation of early survival and cyst number after peroral *Toxoplasma gondii* infection of AxB/BxA recombinant inbred and B10 congenic mice J Immunol 1989 143 3031 3034 2809214

[B15] McLeod R Eisenhauer P Mack D Brown C Filice G Spitalny G Immune responses associated with early survival after peroral infection with *Toxoplasma gondii* J Immunol 1989 142 3247 3255 2496163

[B16] Suzuki Y Yang Q Remington JS Genetic resistance against acute toxoplasmosis depends on the strain of *Toxoplasma gondii* J Parasitol 1995 81 1032 1034 8544050

[B17] Brown CR McLeod R Class I MHC genes and CD8+ T cells determine cyst number in *Toxoplasma gondii* infection J Immunol 1990 145 3438 3441 2121825

[B18] Suzuki Y Joh K Orellana MA Conley FK Remington JS A gene(s) within the H-2D region determines the development of toxoplasmic encephalitis in mice Immunology 1991 74 732 739 1783431 PMC1384788

[B19] Blackwell JM Roberts CW Alexander J Influence of genes within the MHC on mortality and brain cyst development in mice infected with *Toxoplasma gondii*: kinetics of immune regulation in BALB H-2 congenic mice Parasite Immunol 1993 15 317 324 8361774 10.1111/j.1365-3024.1993.tb00616.x

[B20] Brown CR Hunter CA Estes RG Beckmann E Forman J David C Remington JS McLeod R Definitive identification of a gene that confers resistance against *Toxoplasma* cyst burden and encephalitis Immunology 1995 85 419 428 7558130 PMC1383915

[B21] Suzuki Y Orellana MA Schreiber RD Remington JS Interferon-gamma: the major mediator of resistance against *Toxoplasma gondii* Science 1988 240 516 518 3128869 10.1126/science.3128869

[B22] Takács AC Swierzy IJ Lüder CGK Interferon-γ restricts *Toxoplasma gondii* development in murine skeletal muscle cells via nitric oxide production and immunity-related GTPases PLoS One 2012 7 e45440 23024821 10.1371/journal.pone.0045440 PMC3443239

[B23] Pfefferkorn ER Rebhun S Eckel M Characterization of an indoleamine 2,3-dioxygenase induced by gamma-interferon in cultured human fibroblasts J Interferon Res 1986 6 267 279 2427623 10.1089/jir.1986.6.267

[B24] Silva NM Rodrigues CV Santoro MM Reis LF Alvarez-Leite JI Gazzinelli RT Expression of indoleamine 2,3-dioxygenase, tryptophan degradation, and kynurenine formation during in vivo infection with *Toxoplasma gondii*: induction by endogenous gamma interferon and requirement of interferon regulatory factor 1 Infect Immun 2002 70 859 868 11796621 10.1128/iai.70.2.859-868.2002 PMC127654

[B25] Fujigaki S Saito K Takemura M Maekawa N Yamada Y Wada H Seishima M L-tryptophan-L-kynurenine pathway metabolism accelerated by *Toxoplasma gondii* infection is abolished in gamma interferon-gene-deficient mice: cross-regulation between inducible nitric oxide synthase and indoleamine-2,3-dioxygenase Infect Immun 2002 70 779 786 11796611 10.1128/iai.70.2.779-786.2002 PMC127656

[B26] Tenhunen R Marver HS Schmid R Microsomal heme oxygenase J Biol Chem 1969 224 6388 6394 4390967

[B27] Soares MP Bach FH Heme oxygenase-1: from biology to therapeutic potential Trends Mol Med 2009 15 50 58 19162549 10.1016/j.molmed.2008.12.004

[B28] Otterbein LE Choi AMK Heme oxygenase: colors of defense against cellular stress Am J Physiol Lung Cell Mol Physiol 2000 279 L1029 1037 11076792 10.1152/ajplung.2000.279.6.L1029

[B29] Wagener FA Volk HD Willis D Abraham NG Soares MP Adema GJ Figdor CG Different faces of the heme-heme oxygenase system in inflammation Pharmacol Rev 2003 55 551 571 12869663 10.1124/pr.55.3.5

[B30] Ryter SW Alam J Choi AMK Heme oxygenase-1/carbon monoxide: from basic science to therapeutic applications Physiol Rev 2006 86 583 650 16601269 10.1152/physrev.00011.2005

[B31] Soares MP Marguti I Cunha A Larsen R Immunoregulatory effects of HO-1: how does it work? Curr Opin Pharmacol 2009 9 482 489 19586801 10.1016/j.coph.2009.05.008

[B32] Gozzelino R Jeney V Soares MP Mechanisms of cell protection by heme oxygenase-1 Annu Rev Pharmacol Toxicol 2010 50 323 354 20055707 10.1146/annurev.pharmtox.010909.105600

[B33] Otterbein LE Bach FH Alam J Soares M Tao Lu H Wysk M Davis RJ Flavell RA Choi AM Carbon monoxide has anti-inflammatory effects involving the mitogen-activated protein kinase pathway Nat Med 2000 6 422 428 10742149 10.1038/74680

[B34] Kapturczak MH Wasserfall C Brusko T Campbell-Thompson M Ellis TM Atkinson MA Agarwal A Heme oxygenase-1 modulates early inflammatory responses: evidence from the heme oxygenase-1-deficient mouse Am J Pathol 2004 165 1045 1053 15331427 10.1016/S0002-9440(10)63365-2 PMC1618611

[B35] Zuckerbraun BS Billiar TR Otterbein SL Kim PKM Liu F Choi AMK Bach FH Otterbein LE Carbon monoxide protects against liver failure through nitric oxide-induced heme oxygenase 1 J Exp Med 2003 198 1707 1716 14657222 10.1084/jem.20031003 PMC2194127

[B36] Tsoyi K Lee TY Lee YS Kim HJ Seo HG Lee JH Chang KC Heme-oxygenase-1 induction and carbon monoxide- releasing molecule inhibit lipopolysaccharide (LPS)-induced high-mobility group box 1 release in vitro and improve survival of mice in LPS- and cecal ligation and puncture- induced sepsis model in vivo Mol Pharmacol 2009 76 173 182 19366789 10.1124/mol.109.055137

[B37] Lee MY Seo CS Lee JA Lee NH Kim JH Ha H Zheng MS Son JK Shin HK Anti-asthmatic effects of *Angelica dahurica* against ovalbumin-induced airway inflammation via upregulation of heme oxygenase-1 Food Chem Toxicol 2011 49 829 837 21146576 10.1016/j.fct.2010.12.004

[B38] Zhu Z Wilson AT Mathahs MM Wen F Brown KE Luxon BA Schmidt WN Heme oxygenase-1 suppresses hepatitis C vírus replication and increases resistance of hepatocytes to oxidant injury Hepatology 2008 48 1430 1439 18972446 10.1002/hep.22491 PMC2587102

[B39] Pamplona A Ferreira A Balla J Jeney V Balla G Epiphanio S Chora A Rodrigues CD Gregoire IP Cunha-Rodrigues M Portugal S Soares MP Mota MM Heme oxygenase-1 and carbon monoxide suppress the pathogenesis of experimental cerebral malaria Nat Med 2007 13 703 710 17496899 10.1038/nm1586

[B40] Epiphanio S Mikolajczak SA Gonçalves LA Pamplona A Portugal S Albuquerque S Goldberg M Rebelo S Anderson DG Akinc A Vornlocher HP Kappe SH Soares MP Mota MM Heme oxygenase-1 is an anti-inflammatory host factor that promotes murine *Plasmodium* liver infection Cell Host Microbe 2008 3 331 338 18474360 10.1016/j.chom.2008.04.003

[B41] Pham NH Mouriz J Kima PE *Leishmania pifanoi* amastigotes avoid macrophage production of superoxide by inducing heme degradation Infect Immun 2005 73 8322 8233 16299330 10.1128/IAI.73.12.8322-8333.2005 PMC1307057

[B42] Paiva CN Feijó DF Dutra FF Carneiro VC Freitas GB Alves LS Mesquita J Fortes GB Figueiredo RT Souza HS Fantappié MR Lannes-Vieira J Bozza MT Oxidative stress fuels *Trypanosoma cruzi* infection in mice J Clin Invest 2012 122 2531 2542 22728935 10.1172/JCI58525 PMC3386808

[B43] Fux B Rodrigues CV Portela RW Silva NM Su C Sibley D Vitor RW Gazzinelli RT Role of cytokines and major histocompatibility complex restriction in mouse resistance to infection with a natural recombinant strain (type I-III) of *Toxoplasma gondii* Infect Immun 2003 71 6392 6401 14573660 10.1128/IAI.71.11.6392-6401.2003 PMC219541

[B44] Benevides L Milanezi CM Yamauchi LM Benjamim CF Silva JS Silva NM CCR2 receptor is essential to activate microbicidal mechanisms to control *Toxoplasma gondii* infection in the central nervous system Am J Pathol 2008 173 741 751 18688032 10.2353/ajpath.2008.080129 PMC2527091

[B45] Coutinho LB Gomes AO Araújo EC Barenco PV Santos JL Caixeta DR Silva DA Cunha-Júnior JP Ferro EA Silva NM The impaired pregnancy outcome in murine congenital toxoplasmosis is associated with a pro-inflammatory immune response, but not correlated with decidual inducible nitric oxide synthase expression Int J Parasitol 2012 42 341 352 22366549 10.1016/j.ijpara.2012.01.006

[B46] Bartley PM Wright S Sales J Chianini F Buxton D Innes EA Long-term passage of tachyzoites in tissue culture can attenuate virulence of *Neospora caninum* in vivo Parasitology 2006 135 421 432 16762097 10.1017/S0031182006000539

[B47] Silva NM Manzan RM Carneiro WP Milanezi CM Silva JS Ferro EA Mineo JR *Toxoplasma gondii*: the severity of toxoplasmic encephalitis in C57BL/6 mice is associated with increased ALCAM and VCAM-1 expression in the central nervous system and higher blood-brain barrier permeability Exp Parasitol 2010 126 167 177 20434443 10.1016/j.exppara.2010.04.019

[B48] Bradford MM A rapid and sensitive method for the quantitation of microgram quantities of protein utilizing the principle of protein-dye binding Anal Biochem 1976 72 248 254 942051 10.1016/0003-2697(76)90527-3

[B49] Teo CF Zhou XW Bogyo M Carruthers VB Cysteine protease inhibitors block *Toxoplasma gondii* microneme secretion and cell invasion Antimicrob Agents Chemother 2007 51 679 688 17145790 10.1128/AAC.01059-06 PMC1797762

[B50] Luz NF Andrade BB Feijó DF Araújo-Santos T Carvalho GQ Andrade D Abánades DR Melo EV Silva AM Brodskyn CI Barral-Netto M Barral A Soares RP Almeida RP Bozza MT Borges VM Heme oxygenase-1 promotes the persistence of *Leishmania chagasi* infection J Immunol 2012 188 4460 4467 22461696 10.4049/jimmunol.1103072 PMC3331931

[B51] Seixas E Gozzelino R Chora A Ferreira A Silva G Larsen R Rebelo S Penido C Smith NR Coutinho A Soares MP Heme oxygenase-1 affords protection against noncerebral forms of severe malaria Proc Natl Acad Sci USA 2009 106 15837 15842 19706490 10.1073/pnas.0903419106 PMC2728109

[B52] Dunay IR Damatta RA Fux B Presti R Greco S Colonna M Sibley LD Gr1(+) inflammatory monocytes are required for mucosal resistance to the pathogen *Toxoplasma gondii* Immunity 2008 29 306 317 18691912 10.1016/j.immuni.2008.05.019 PMC2605393

[B53] Otterbein LE Soares MP Yamashita K Bach FH Heme oxygenase-1: unleashing the protective properties of heme Trends Immunol 2003 24 449 455 12909459 10.1016/s1471-4906(03)00181-9

[B54] Wagner A Förster-Waldl E Garner-Spitzer E Schabussova I Kundi M Pollak A Scheiner O Joachim A Wiedermann U Immunoregulation by *Toxoplasma gondii* infection prevents allergic immune responses in mice Int J Parasitol 2009 39 465 472 18938169 10.1016/j.ijpara.2008.09.003

[B55] Morampudi V De Craeye S Le Moine A Detienne S Braun MY D’Souza S Partial depletion of CD4(^+^)CD25(^+^)Foxp3(^+^) T regulatory cells significantly increases morbidity during acute phase *Toxoplasma gondii* infection in resistant BALB/c mice Microbes Infect 2011 13 394 404 21262371 10.1016/j.micinf.2011.01.006

[B56] Khan IA Matsuura T Kasper LH IL-10 mediates immunosuppression following primary infection with *Toxoplasma gondii* in mice Parasite Immunol 1995 17 185 195 7624159 10.1111/j.1365-3024.1995.tb00888.x

[B57] Yamashita K Ollinger R McDaid J Sakahama H Wang H Tyagi S Csizmadia E Smith NR Soares MP Bach FH Heme oxygenase-1 is essential for and promotes tolerance to transplanted organs FASEB J 2006 20 776 778 16473885 10.1096/fj.05-4791fje

[B58] Jung ID Lee JS Lee CM Noh KT Jeong YI Park WS Chun SH Jeong SK Park JW Son KH Heo DR Lee MG Shin YK Kim HW Yun CH Park YM Induction of indoleamine 2,3-dioxygenase expression via heme oxygenase-1-dependant pathway during murine dendritic cell maturation Biochem Pharmacol 2010 80 491 505 20430013 10.1016/j.bcp.2010.04.025

[B59] Cerávolo IP Chaves AC Bonjardim CA Sibley D Romanha AJ Gazzinelli RT Replication of *Toxoplasma gondii*, but not *Trypanosoma cruzi*, is regulated in human fibroblasts activated with gamma interferon: requirement of a functional JAK/STAT pathway Infect Immun 1999 67 2233 2240 10225879 10.1128/iai.67.5.2233-2240.1999 PMC115962

[B60] Chaves AC Cerávolo IP Gomes JA Zani CL Romanha AJ Gazzinelli RT IL-4 and IL-13 regulate the induction of indoleamine 2,3-dioxygenase activity and the control of *Toxoplasma gondii* replication in human fibroblasts activated with IFN-gamma Eur J Immunol 2001 31 333 344 11180096 10.1002/1521-4141(200102)31:2<333::aid-immu333>3.0.co;2-x

[B61] Divanovic S Sawtell NM Trompette A Warning JI Dias A Cooper AM Yap GS Arditi M Shimada K Duhadaway JB Prendergast GC Basaraba RJ Mellor AL Munn DH Aliberti J Karp CL Opposing biological functions of tryptophan catabolizing enzymes during intracellular infection J Infect Dis 2012 205 152 161 21990421 10.1093/infdis/jir621 PMC3242739

[B62] Maines MD Zinc protoporphyrin is a selective inhibitor of heme oxygenase activity in the neonatal rat Biochim Biophys Acta 1981 673 339 350 6894392 10.1016/0304-4165(81)90465-7

[B63] Cherayil BJ Indoleamine 2,3-dioxygenase in intestinal immunity and inflammation Inflamm Bowel Dis 2009 15 1391 1396 19322906 10.1002/ibd.20910

[B64] Harrington L Srikanth CV Antony R Rhee SJ Mellor AL Shi HN Cherayil BJ Deficiency of indoleamine 2,3-dioxygenase enhances commensal-induced antibody responses and protects against *Citrobacter rodentium*-induced colitis Infect Immun 2008 76 3045 3053 18426872 10.1128/IAI.00193-08 PMC2446721

