# Supplementary figures and images for: Heme oxygenase-1 activity is involved in the control of Toxoplasma gondii infection in the lung of BALB/c and C57BL/6 and in the small intestine of C57BL/6 mice
Source: Vet Res. 2013 Oct 2;44(1):89. doi: 10.1186/1297-9716-44-89 (PMC3851451; doi:10.1186/1297-9716-44-89)

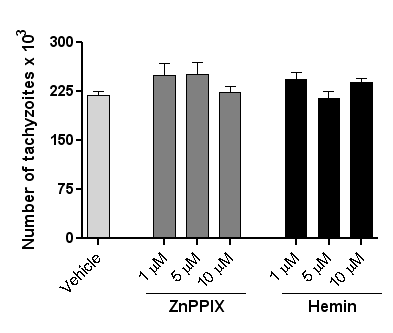

Supplement: Additional file 1 — T gondii intracellular proliferation in HeLa cells after parasite treatment with ZnPPIX, hemin or vehicle. Tachyzoites of T. gondii 2 F1 strain were treated for 1 h with different concentrations of ZnPPIX or hemin or vehicle. After 24 h of infection, the experiment was analyzed for T. gondii intracellular proliferation determined by a colorimetric microtiter assay using β-galactosidase-expressing tachyzoites. Data are expressed as mean ± SD of the number of tachyzoites calculated in relation to a reference curve and are representative of two independent experiments performed in quadruplicate. [file 1297-9716-44-89-S1.TIF]
